# Feasibility of mitigating out-toeing gait using compression tights with inward-directing taping lines

**DOI:** 10.1371/journal.pone.0291914

**Published:** 2023-09-21

**Authors:** Prabhat Pathak, Hye Suk Kim, Hyunji Kim, Juyeon Park, Giuk Lee, Jooeun Ahn

**Affiliations:** 1 John A. Paulson School of Engineering and Applied Sciences, Harvard University, Cambridge, MA, United States of America; 2 Research Institute of Human Ecology, Seoul National University, Seoul, Republic of Korea; 3 Department of Physical Education, Seoul National University, Seoul, Republic of Korea; 4 Soft Robotics Research Center, Seoul National University, Seoul, Republic of Korea; 5 Department of Fashion and Textiles, Seoul National University, Seoul, Republic of Korea; 6 Department of Mechanical Engineering, Chung-Ang University, Seoul, Republic of Korea; 7 Institute of Sport Science, Seoul National University, Seoul, Republic of Korea; Delft University of Technology, NETHERLANDS

## Abstract

Out-toeing gait may cause alterations in lower limb biomechanics that could lead to an increased risk of overuse injuries. Surgery and physical therapy are conventional methods for mitigating such gait, but they are costly and time-consuming. Wearable devices like braces and orthoses are used as affordable alternatives, but they apply non-negligible stress on the skin. Haptic feedback-delivering shoes were also recently developed, but they require actuators and power sources. The purpose of our study is to develop compression tights with inward directing taping lines that apply compression to lower limb muscles and segments to facilitate inward rotation of the foot, overcoming the drawbacks of previous methods. These compression tights were manufactured to fit the average height, leg length, hip girth, and waist girth of South Korean females in their twenties. The efficacy of these compression tights was evaluated by comparing walking kinematics and user satisfaction of 12 female dancers with an out-toeing gait under three conditions: wearing tights with taping lines, tights without taping lines, and basic bicycle shorts. The foot rotation angles and joint kinematics were recorded using a pressure-pad treadmill and motion capture system, respectively. Multiple pairwise comparisons revealed that the compression tights with inward-directing lines significantly reduced foot rotation angles (up to an average of 20.1%) compared with the bicycle shorts (p = 0.002 and 0.001 for dominant and non-dominant foot, respectively) or the compression tights without taping lines (p = 0.005 and p = 0.001 for dominant and non-dominant foot, respectively). Statistical parametric mapping revealed significant main effects of the tight type on joint kinematics. Also, t-tests revealed that the participants reported significantly higher ratings of perceived functionality and usability on the compression tights with inward-directing taping lines. In conclusion, we developed a comfortable and practical apparel-type wearable and demonstrated its short-term efficacy in mitigating out-toeing gait.

## Introduction

Out-toeing gait is a potentially detrimental rotational walking deformity that is common among patients with osteoarthritis, children with cerebral palsy [1, 2], and even individuals stereotypically viewed as healthy, such as infants and dancers [3, 4]. Walking with a large foot rotation or progression angle is associated with a broad profile of lower limb deformities, which commonly include decreased range of motion, hip external rotation contracture, external tibial torsion, and femoral retroversion [3, 5, 6]. These deformities frequently apply excessive stress on the lower limb tendons, ligaments, and joints, thereby increasing the risk of falls, fractures, and other injuries associated with lower limb overuse [7–9].

Numerous methods have been proposed to mitigate out-toeing gait. Customized physical therapy, gait retraining exercises, and rotational gait corrective surgeries have been considered as conventional methods for treating such gait [10–12], but these methods are time-consuming and costly. Therefore, wearable approaches targeting the distal joints have been proposed as a relatively acute and inexpensive option. Ankle braces, ankle knee orthoses, and shoes embedded with vibrating actuators delivering haptic feedback to the feet have been developed to reduce foot rotation angles [13–15]. Although effective, braces and orthoses apply non-negligible mechanical stress on the skin and reduce the range of motion [16], possibly disrupting walking and daily activities. The vibrating shoes are also functional, but they require actuators and power sources. In addition, long-term exposure to vibration can diminish human motor performance by impeding the function of muscular, proprioceptive, and somatosensory systems [17, 18].

In this study, we aimed to devise an effective, comfortable, passive, and light wearable approach that may benefit individuals who wish to mitigate their out-toeing gait without decreasing their motor performance. To this end, we developed compression tights with inward-directing taping lines targeting multiple lower limb segments that affect the foot rotation angle [19, 20]. This concept was motivated by studies that showed the efficacy of segment-targeted direct skin taping and compression garments. Song et al. showed that tape applied on the thighs to facilitate inward femoral rotation alleviated pain, improved postural stability, and reduced detrimental joint movements that lead to overuse injuries in patients with patellofemoral pain [21, 22]. However, Kinesio tape adhesion can cause skin irritation, making it uncomfortable for daily use [23]. We attempted to resolve this by embedding taping lines into compression tights. Previous studies have shown that tights can also enhance running economy [24, 25], attenuate soft-tissue vibration [26, 27], and improve jump performance [28, 29]. Multiple studies also report that tights can enhance performance and mitigate injuries during manual labor by applying proper compression to specific muscles and segments [30, 31]. These studies proposed that wearing compression garments designed for adequate anatomical support can mechanically calibrate the contraction direction of muscle fibers, reduce performance-impeding muscle displacement, and facilitate efficient movement.

The purpose of this study is to develop compression tights with inward-directing taping lines for mitigating out-toeing gait and assess the efficacy of the developed tights. We designed and manufactured tights with inward-directing taping lines, and recruited female dancers, who typically walk with large foot rotation angles owing to their training regimen, which requires extension of the feet up to the extrema of the range of motion [4, 7, 32]. We hypothesized that wearing compression tights with inward-directing taping lines would reduce the foot rotation angle during walking. We also hypothesized that compression tights with inward-directing taping lines would improve perceived functionality and usability during walking, and formulated a questionnaire to test the hypothesis.

## Materials and methods

### Participants

We recruited 12 college-level female dancers aged between 20 and 29 (mean = 24.7; standard deviation, SD = 2.9) years. We assume that the effect of wearing tights on the primary indicator of out-toeing gait, foot rotation angle, would conservatively have a medium effect size (ES) of 0.5 [33]. We then selected the estimated power and p-value for statistical significance as 0.95 and 0.05, respectively. With these inputs, the G-power software [34] calculated a sample size of 12.

The participants had received professional dance training for 14.1 (SD = 2.0) years, and had no known history of lower limb injury or surgery for at least 6 months at the time of the experiment. We recruited those who met the range of body dimensions considered to represent the average size of Korean females in their 20s, according to data retrieved from the national anthropometric survey [35]. The four key body measurements–height, leg length, waist girth, and hip girth, were considered in the inclusion criteria, based on the standards for clothing size designations [35, 36]. The mean and standard deviations of weight, height, leg length, waist girth, and hip girth for the 12 participants were 51.3 (SD = 4.1) kg, 163.3 (SD = 3.6) cm, 74.9 (SD = 2.2) cm, 65.0 (SD = 3.7) cm, and 91.4 (SD = 4.3) cm, respectively. The dominant limb for each participant was defined as the limb that the participant prefers to use when kicking a ball [37].

We informed the participants about all aspects of the study, as approved by the Seoul National University Institutional Review Board (IRB No. 2008/003-029). All participants were provided with an opportunity to review and sign a consent form before the experiment.

### Instruments

#### Compression tights

**Fig 1:**
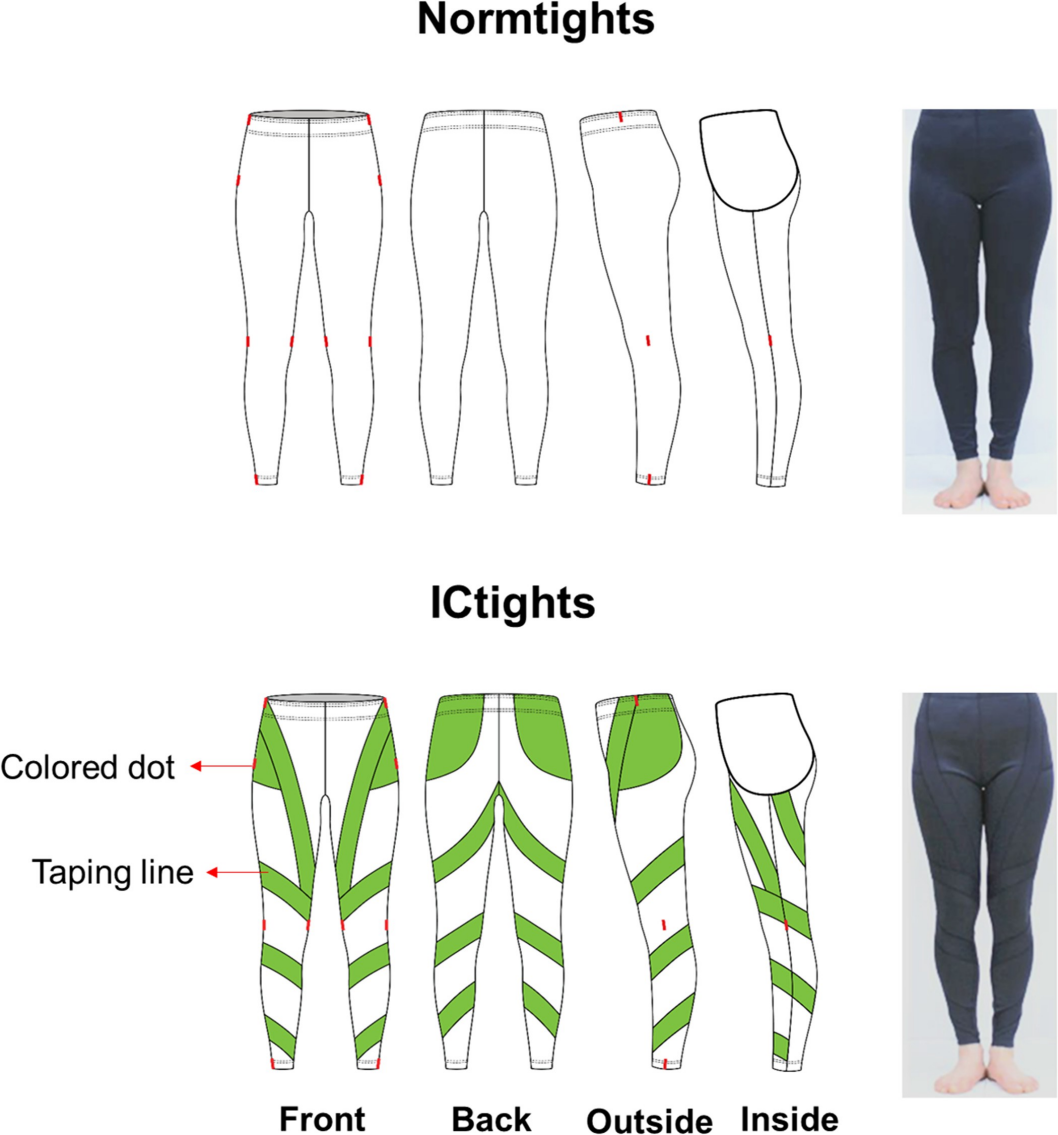
Design of two prototype compression tights. ICtights are embedded with inward-directing taping lines, whereas Normtights are not. The green lines represent the position of the taping lines that apply compression to the target muscles and segments responsible for hip internal rotation, femoral stabilization, and internal tibial rotation. The colored dots are sewed into the anatomical landmarks of the hip, knee, and ankle joints to allow the participants to wear the tights in such a way that the taping lines apply pressure on the intended muscles and segments.

We prototyped two tights in the same silhouette and size (Fig 1): one with inward-directing taping lines (ICtights) and the other without taping lines (Normtights). For the ICtights, one layer of 4 cm wide taping lines, manufactured using power net fabric (nylon/spandex: 80/20), were sewn with flat-lock stitches along the outline. We embedded these taping lines to provide additional sensation for the target muscles and joints and facilitate inward rotation of the feet. The taping lines originated at the waist band and terminated at the ankle cuff. The lines ran over the gluteus, tensor fasciae latae, sartorius, biceps femoris, and semitendinuous muscles to facilitate hip internal rotation and femoral stabilization. The taping line was followed through the ankle joint to ensure inward compression for the internal rotation of the tibia. To achieve these design goals, we manufactured the pattern so that the taping lines with lower elasticity compared to the ground fabric went around the tights 3 times with the inclination angles of 26 and 34 degrees (S1 Fig in S2 File). Colored dots were sewn on the compression tights to allow the participants to wear the tights properly so that the taping lines applied pressure on the target muscles and segments. We also manufactured Normtights to investigate any possible effect of wearing simple tights on foot rotation angles.

We used an elastomer-plated double-jersey fabric (polyester/spandex: 74/26) to manufacture areas of ICtights other than the taping lines and all areas of the Normtights. Based on the average size of Korean females in their 20s, we drafted block patterns for the compression pants following Armstrong’s patternmaking techniques [38]. Both ICtights and Normtights were made in two size designations, small and medium, to cover the range of body measurements of the recruited participants. The different sizes of the tights were determined using the standard sizing and grading procedure [35].

We additionally prepared cycling shorts (Model: TAKE FIVE 022; polyester/spandex: 92/8) with minimal compression that conformed with the body dimensions of the participants (small and medium). These shorts were used as a baseline tights condition (Baseline) to acquire foot rotation angles during walking with minimal pressure application on the lower limbs.

#### Foot rotation and lower limb joint angle acquisition

We recorded the foot rotation angle during walking using a pressure-pad embedded treadmill (Gait analysis FDM-TDSL-3i, Zebris Inc., Germany). The treadmill was also utilized to record the vertical ground reaction force (VGRF), which was used to detect the beginning and end of the stance phase. Ten infrared cameras (Optitrack Prime^X^ 13, Natural Point, Inc., Oregon, USA) were used to record the position of the retroreflective markers attached to the anatomical landmarks of the lower limb, and three-dimensional joint angles were calculated using the position of the markers. The sampling frequencies of the treadmill and cameras were 100 Hz, and the two systems were synchronized using a sync box.

### Perceived functionality and usability

We investigated the usability of the devised ICtights. The International Organization for Standardization defines factors of usability as the user satisfaction, usefulness and the ease of use [39]. To address these factors, we formulated a user questionnaire based on previous studies that assessed the clothing fit [40], comfort of compression garments [41–43], and criteria for usability evaluation [44, 45] with confirmed validity. The questionnaire consisted of seven questions broadly categorized into two sections: perceived functionality and usability of the compression tights during walking. The detailed questions are summarized in S1 Table in S2 File.

### Experimental protocol

We asked each participant to bring in an athletic t-shirt and shoes to put on during the experiment. Before performing the walking task, we measured the body dimensions of the participants using Martin’s anthropometer in an anthropometric standing posture [36]; breathing normally, standing upright, and the arms hanging naturally beside their torso. We asked the participants to wear the baseline shorts and athletic t-shirt while measuring the body dimensions. Then, to measure the preferred walking speed (PWS) of the participants, we asked the participants to walk at 2.5 km/hr initially and increased the speed by 0.1 km/hr every 10 seconds until the participants reported the speed that best characterized their everyday walking speed. We then increased the speed by 1.0 km/hr and decreased it by 0.1 km/hr every 10 seconds until they again reported the speed that best characterized their everyday walking speed [46, 47]. We repeated this process three times, and the average speed was selected as the PWS.

After determining the PWS, we attached 20 retro-reflective markers on the anatomical landmarks of the lower limbs: left and right heel, first and fifth metatarsal, medial and lateral malleolus, medial and lateral epicondyle, greater trochanter, and anterior and posterior iliac spinae (S2 Fig in S2 File). Each participant performed walking trials under three conditions: wearing Baseline, Normtights, and ICtights. We randomized the sequence of the conditions, along with a 10-min break between trials, including the time for changing the attire, and reattach the retro-reflective markers. Only one investigator attached the markers, following the standard anthropometric measurement guidelines [48], for all the participants to maintain consistency in the location of the anatomical landmarks. Considering the usual time required for adaptation to a treadmill system [49], we asked the participants to walk for 10 min in the first trial, and 7 min each in the second and third trials, and analyzed the data acquired in the last 5 min of all the trials. After treadmill walking, the participants rated their experience of Normtights and ICtights using a 5-point Likert scale. They also provided supplementary explanations for their ratings.

### Data analysis

The angle between the longitudinal axis of the foot and the walking direction (the foot rotation angle) was automatically calculated using the treadmill data acquisition software (Zebris FDM 1.12, Zebris Medical GmbH, Germany). Nüesch et al. reported the foot rotation angles acquired from the treadmill we used in our study to have high reliability across multiple walking sessions [50]. The coordinates of the retroreflective markers were filtered using a zero-lag fourth-order Butterworth low-pass filter with a cut-off frequency of 10 Hz. The filtered coordinates of the anatomical landmarks were used to build a seven-segment (left and right foot, shank, and thigh and a pelvis) lower body model utilizing a modeling software (Visual 3D). We calculated the three-dimensional angles of the left and right ankles, knees, and hip joints using the visual 3D internal algorithm and the seven-segment model. The coordinate system for the sagittal, frontal, and transverse plane joint angles followed the Cardan sequence, which was defined as flexion (+)/extension (–), adduction (+)/abduction (–), and internal (+)/external rotation (–), respectively. We extracted the joint angles during the stance phase of each leg using the VGRF curves. The start and end of the stance phase were selected as heel strike and toe off, which we respectively defined as first and last time points of the VGRF curves. We analyzed the three-dimensional lower limb joint angles over the normalized stance between 0 and 100% (101 points).

### Statistical analysis

We performed one-way repeated measures analysis of variance (ANOVA) to compare the foot rotation angle separately for each limb, under the three conditions (Baseline, Normtights, and ICtights). Mauchly’s test was conducted to assess the assumption of sphericity. If this was violated, we reduced the degrees of freedom using the Greenhouse-Geisser criterion. Bonferroni correction was used as a post-hoc test for multiple pairwise comparisons. We also performed statistical parametric mapping (SPM) and one-way repeated measures ANOVA to compare the three-dimensional hip, knee, and ankle joint angles over the stance phase separately for each limb under the three conditions.

The ratings reported by the participants during the user questionnaire were tested for normality using the Shapiro–Wilk test. If the normality assumption was accepted, a paired t-test was used to compare differences in ratings for perceived functionality and usability between the Normtights and ICtights. If the assumption was rejected, the Wilcoxon-signed rank test was used as an alternative to the paired t-test.

Statistical analyses to compare foot rotation angles and ratings gathered during the user questionnaire were conducted using Statistical Package for the Social Sciences (version 25.0, International Business Machines Inc., Armonk, NY, USA). SPM was conducted using MATLAB 2018b (version 2018b, Mathworks Inc., Natick, MA, USA) and the open-source code available at https://spm1d.org/. The level of statistical significance was set at p < 0.05.

## Results

### Foot rotation angle

**Fig 2:**
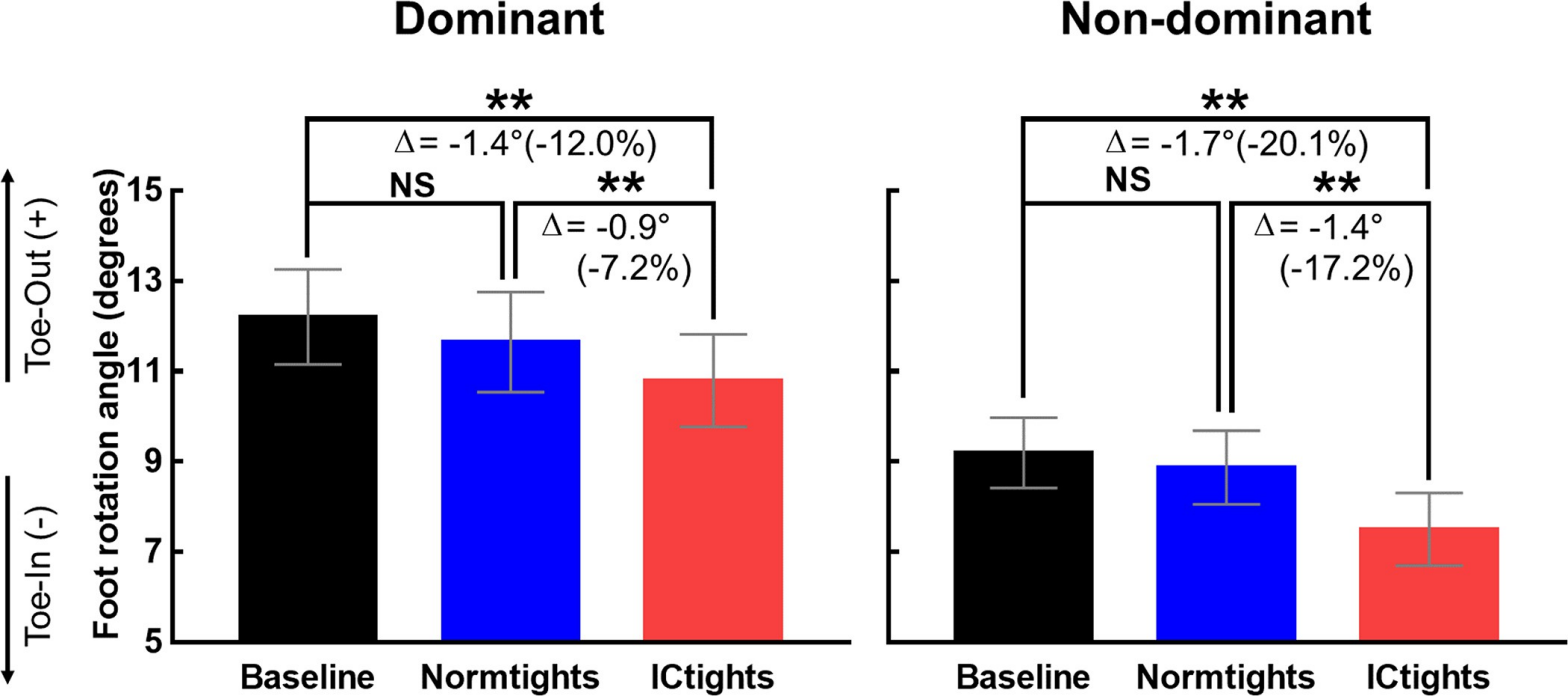
Foot rotation angles in the three tights type conditions. Mean and standard error bars of the foot rotation angles of 12 participants for the three conditions (Baseline: bicycle shorts, Normtights: compression tights without taping lines, and ICtights: compression tights embedded with taping lines). The double asterisk indicates a statistically significant difference (**p < 0.01), whereas NS indicates no statistically significant difference. The reduction in the foot rotation angle while walking wearing the ICtights with respect to the Baseline or Normtights is quantified as Δ (%).

The mean and standard errors of the foot (left and right) rotation angles of all the participants under the three experimental conditions are shown in Fig 2. The results of one-way repeated measures ANOVA revealed a significant main effect of the tight type on the foot rotation angles for both feet (dominant: F_[__2__,_ _22__]_ = 12.707, p < 0.001, *η*^*2*^ = 0.536, ES = 1.075; non-dominant: F_[__2__,_ _22__]_ = 16.993, p < 0.001, *η*^*2*^ = 0.607, ES = 1.243). ICtights induced significantly lower foot rotation angles than those of the Baseline (dominant: p = 0.002, non-dominant: p = 0.001); and Normtights (dominant: p = 0.005, non-dominant: p = 0.001). The reduction in the foot rotation angle under the ICtights condition with respect to the angle under the other two conditions is indicated as Δ (%) in Fig 2. The average Δ values were between 0.9°(7.2%) and 1.7°(20.1%).

### Three-dimensional joint angles

**Fig 3:**
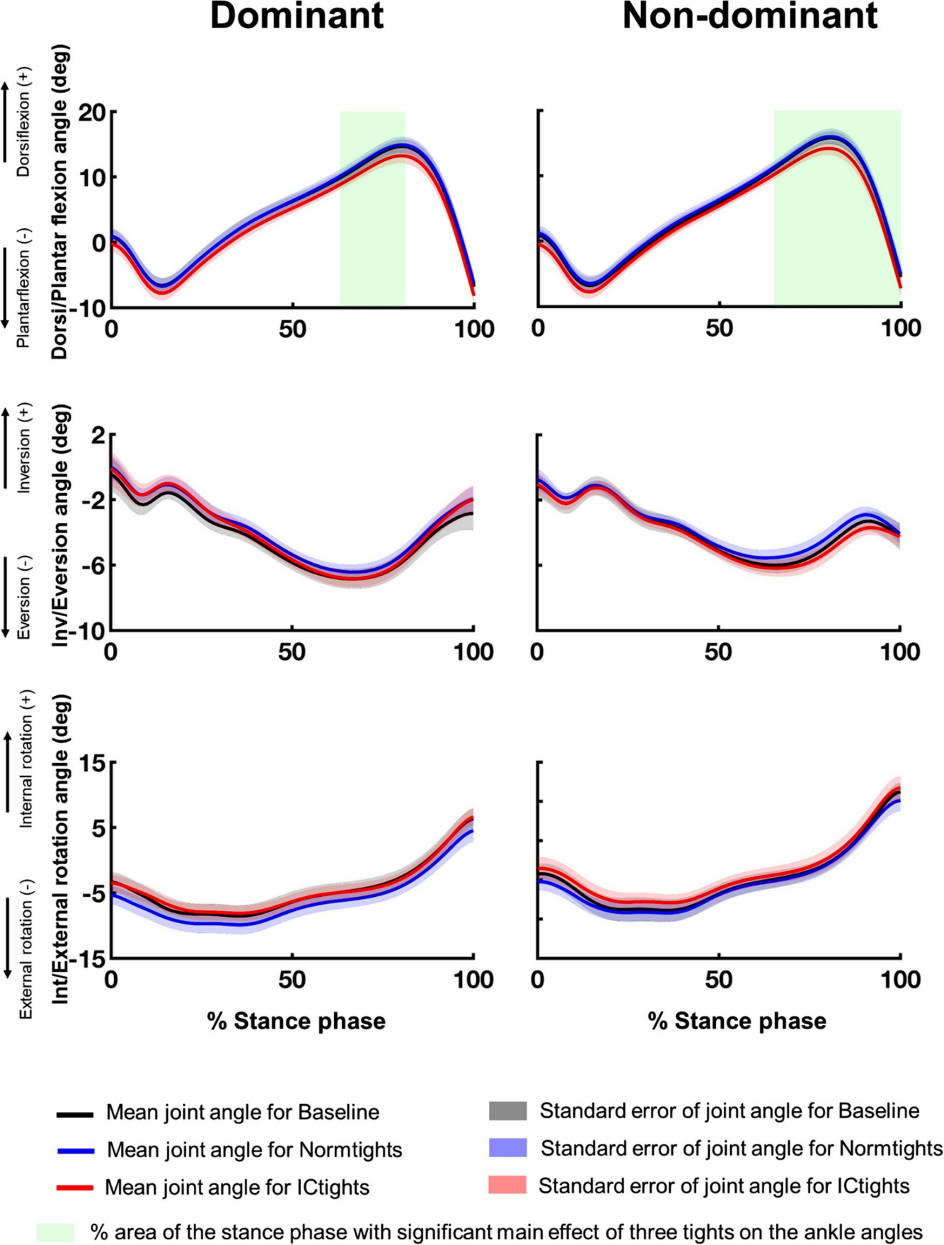
Three-dimensional ankle joint angles during the stance phase in the three tights type conditions. Mean and standard errors of the three-dimensional ankle joint angles of both limbs: dorsiflexion (+)/plantarflexion (–), inversion (+)/eversion (–), and internal rotation (+)/external rotation (–) of the 12 participants while walking wearing the three types of tights. The black, blue, and red lines are the mean ankle angles of the 12 participants under Baseline, Normtights, and ICtights conditions, respectively, and the shaded areas in respective colors show the standard errors of the ankle angles during the stance phase. The green shaded area corresponds to the percentage of the stance phase with a significant main effect of tights on the ankle angles.

**Fig 4:**
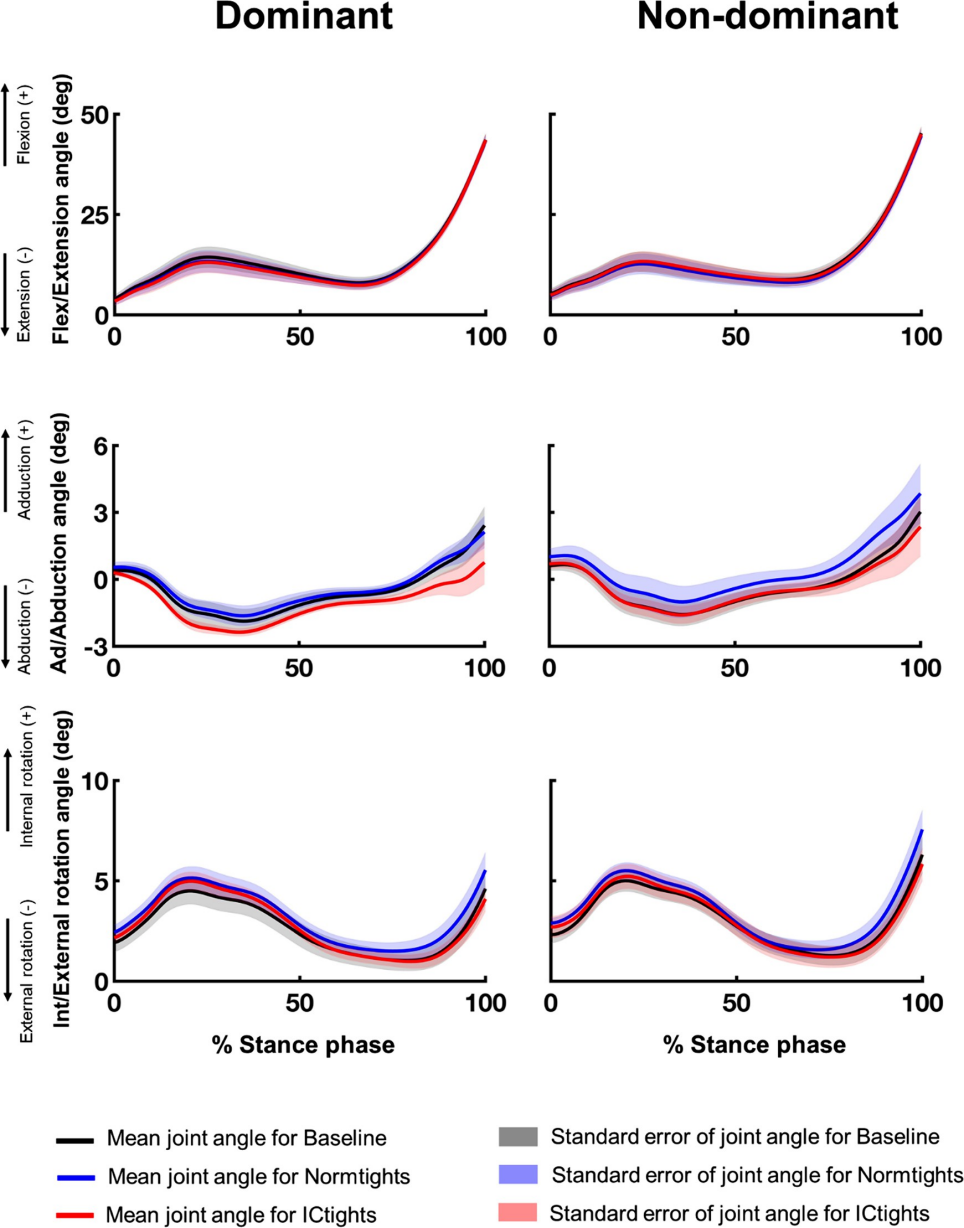
Three-dimensional knee joint angles during the stance phase in the three tights type conditions. Mean and standard error bars of the three-dimensional knee joint angles of both limbs: flexion (+)/extension(–), adduction (+)/abduction (–), and internal rotation (+)/external rotation (–) of the 12 participants while walking wearing the three types of tights. The black, blue, and red lines are the mean knee angles of the 12 participants under Baseline, Normtights, and ICtights conditions, respectively, and the shaded areas in respective colors show the standard errors of the knee angles during the stance phase.

**Fig 5:**
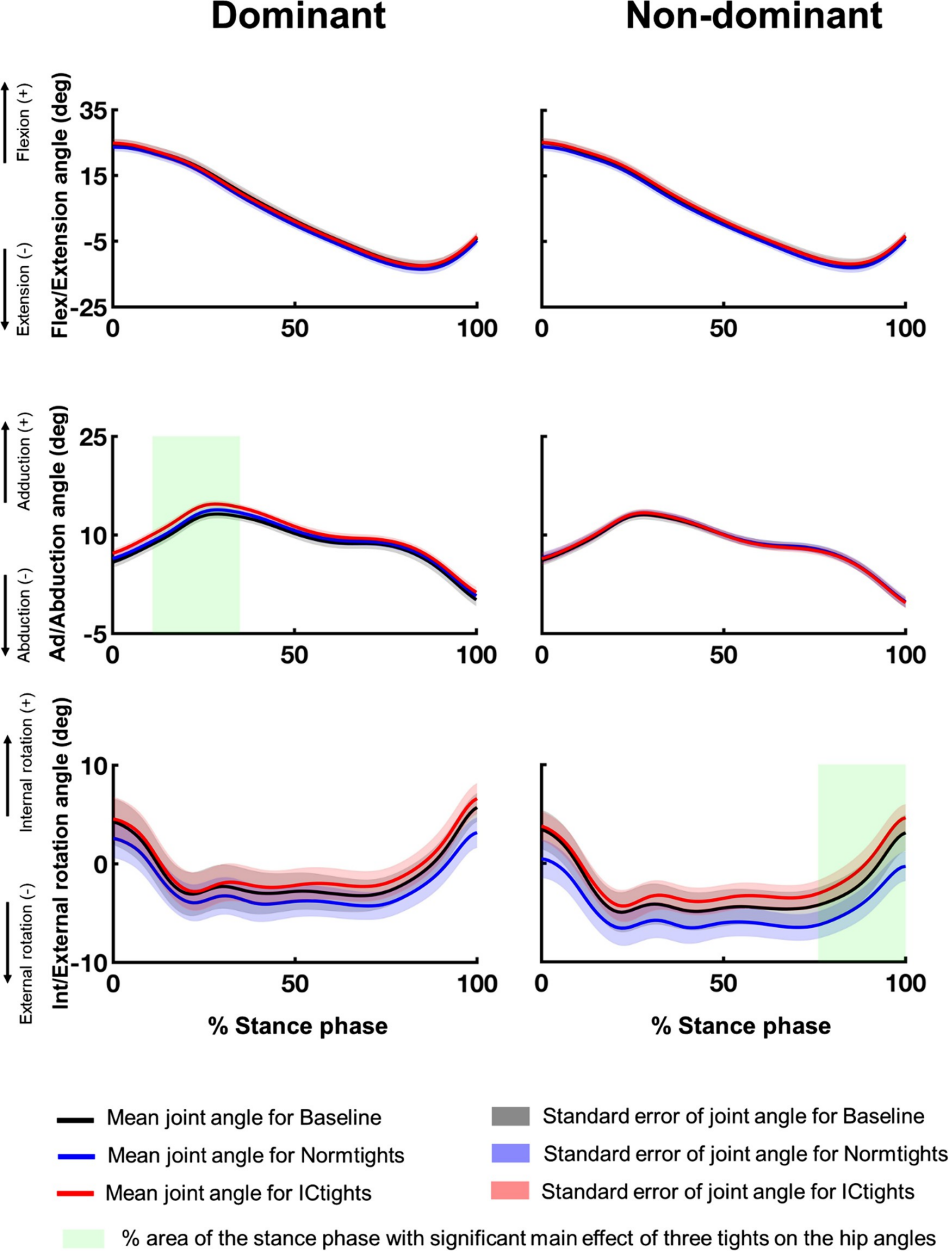
The three-dimensional hip joint angles during the stance phase in the three tights type conditions. Mean and standard error bars of the three-dimensional hip joint angles of both limbs: flexion (+)/extension (–), adduction (+)/abduction (–), and internal rotation (+)/external rotation (–) of the 12 participants while walking wearing the three types of tights. The black, blue, and red lines are the mean hip angles of the 12 participants under Baseline, Normtights, and ICtights conditions, respectively, and the shaded areas in respective colors show the standard errors of the hip angles during the stance phase. The green shaded area corresponds to the percentage during the stance phase with a significant main effect of tights on the hip angles.

Figs 3–5 show the mean and standard error of the three-dimensional lower limb joint angles of the 12 participants during the stance phase of walking under the three conditions. The results of the main effect of one-way repeated measures ANOVA are shown in S3 to S5 Figs in S2 File. The significant main effects of the tight type on joint kinematics were revealed for ankle dorsi/plantarflexion angles for both limbs (dominant: between 63 and 81% of stance phase, p = 0.020; non-dominant: between 65 and 100% of stance phase, p = 0.002), hip adduction/abduction angles for the dominant limb (between 11 and 35% of stance phase, p = 0.017), and hip internal/external rotation angles for the non-dominant limb(between 76 and 100% of stance phase, p = 0.025).

### Perceived functionality and usability

**Fig 6:**
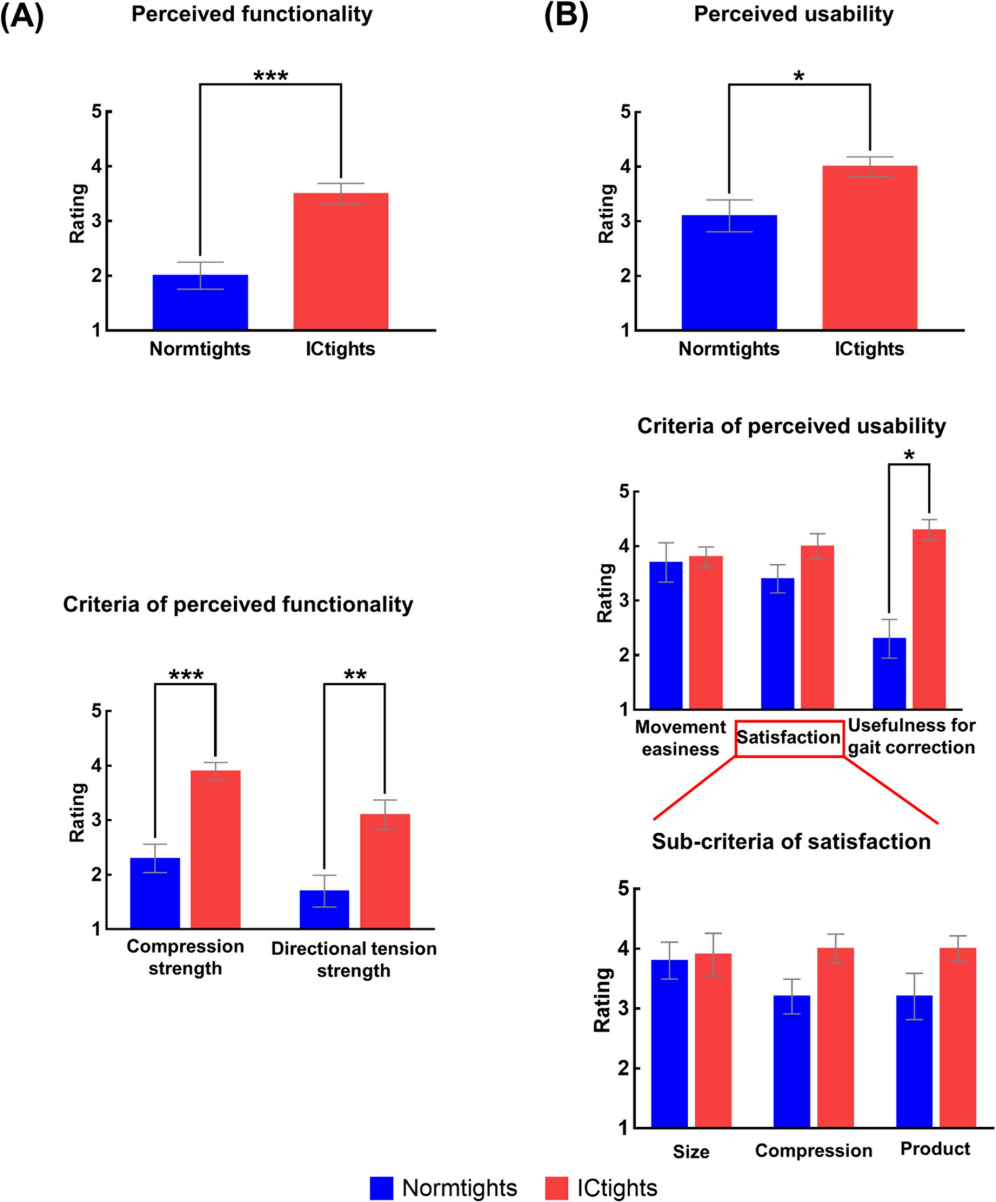
Perceived functionality and usability for the two prototype compression tights. Mean and standard error bars of the ratings for perceived functionality and usability, and separately for their respective criteria, for the two compression tights, as reported by 12 participants (Normtights: compression tights without taping lines, and ICtights: compression tights embedded with taping lines). The asterisks indicate statistically significant differences: ***p < 0.001, **p < 0.01, and *p < 0.05.

Fig 6 shows the mean and standard error of the ratings reported by the participants for all criteria of perceived functionality and usability. The results from the paired t-test or Wilcoxon signed-rank test are presented in S3 Table in S2 File. The participants reported significantly higher ratings for ICtights than Normtights for all the criteria of perceived functionality. For perceived usability, the participants reported significantly higher ratings for ICtights than Normtights for usefulness in gait correction. Although no statistical significance was observed in the other criteria of perceived usability, the average ratings of ICtights were all higher than those of Normtights. The supplementary comments provided by the participants are shown in S1 Results in S2 File. The reliability analysis of the items used in the questionnaire resulted in a Cronbach’s alpha value of 0.93.

## Discussion

We developed compression tights with inward-directing taping lines and assessed their short-term efficacy in correcting out-toeing gait of young adult female dancers. We found that wearing the compression tights reduced the foot rotation angle by up to an average of 20%. Participants walked with larger hip adduction, larger internal rotation angles for all the lower limb joints, and larger plantar flexion of the ankle joint when wearing the devised tights compared to the cases of wearing tights without taping lines and bicycle shorts. They also reported high perceived functionality and usability of the tights. These results suggest that for female dancers, wearing compression tights with inward-directing taping lines effectively mitigates out-toeing gait in the short-term without compromising user satisfaction.

One distinctive feature of the devised tights is the insertion of the taping lines. Direct taping can cause irritation [23], and we resolved this by embedding the taping lines on the compression tights without adhesion. Effectively, no participant reported irritation or discomfort due to skin contact with the material used to manufacture the tights or added pressure from the taping lines; the taping lines embedded in the tights applied additional sensation to joints and muscles to facilitate inward rotation of the foot without discomfort.

We also considered that the efficacy of Kinesio tapes heavily depends on the taping technique; the tapes should be applied to anatomically accurate positions and in the proper directions. To minimize any possible decrease in efficacy due to inadequate alignment between the embedded taping lines and the target muscles, we sewed colored dots onto the compression tights, specifically on the anatomical landmarks for the ankle, knee, and hip joints (Fig 1). The presence of these dots, which is another distinctive feature of the devised tights, guided the participants on how to appropriately wear the compression tights so that the taping lines could apply the intended pressure only to the target regions.

Another feature that distinguishes the devised tights from previous methods for out-toeing correction is that these tights are designed to influence proximal joints and limbs as well as distal ones. Cui et al. reported that the primary kinematic changes observed in the out-toeing gait not only include restrictive ankle plantar flexion but also the overall external rotation of the ankle, knee, and hip joints [19]. Specifically among dancers, excessive foot outward rotation training from an early age leads to torsional deformity of the lower limbs, such as femoral retroversion and lower limb external torsion [51–53]. We showed that wearing compression tights with inward-directing taping lines led to an overall increase in ankle plantar flexion angles and internal rotation angles of all three lower limb joints throughout the stance phase (Figs 3 to 5); the devised compression tights successfully involved the entire lower limb kinematic chain in mitigating out-toeing gait beyond just changing foot kinematics. This design may also aid in decreasing discomfort by distributing the mechanical stress due to the intervention across the relatively larger parts of the lower limbs and joints, rather than applying all the stress solely on the distal joints and limbs. The high usability reported by the participants supports this argument.

Although Thielen et al. showed the efficacy of applying rotation bandages to the lower limb in changing foot rotation angles [54], our study is the first to propose the use of compression garments embedded with taping lines to correct rotational deformities. Most previous methods used to correct out-toeing gait are either time-consuming, expensive, impeding motor performance, or requiring active power sources. The method we propose is to wear compression tights, which can be a part of everyday apparel. The compression tights required neither expensive materials nor a special manufacturing process, yet showed immediate effects without hampering comfort or perceived walking functionality. The supplementary comments provided by the participants also confirmed the user’s perception of improved balance and overall inward rotation of the thighs and calves during walking (S1 Results in S2 File). Although the compression tights we developed are effective, comfortable and inexpensive, the effect of the tights on out-toeing mitigation might be different from that of other available methods. Hence, a future study may consider comparing the effect size of the change in foot rotation angles, comfortability, and usability of other methods with those of the tights developed in this study.

Several other limitations of our study also need to be clarified. First, we assessed the feasibility of the devised intervention by quantifying the effect of the tights on female adult dancers with an out-toeing gait. This specific group was deliberately chosen in this initial study to minimize any possible risk and facilitate the study process. Further studies are necessary to confirm the beneficial effects of the devised intervention on other groups, such as patients with osteoarthritis and children with cerebral palsy. Second, we designed the tights to apply pressure to the muscles and segments that facilitate the reduction of foot rotation angles during walking. Future studies are necessary to confirm the effect of the proposed tights in reducing foot rotation angles during motor tasks other than walking. Third, we did not measure the exact pressure applied by the compression tights. Instead, we manufactured the tights to fit the mean and one standard deviation range of body dimensions for Korean females in their 20s. Considering that the range of the tension exerted by compression tights is 175~350 N/m [55], Laplace’s law predicts the range of the garment pressure applied to the thigh and calf as 15~30 mmHg and 24~48 mmHg, respectively [56]. However, prior studies have reported that a single-pressure level for compression garments is most beneficial for enhancing exercise performance [57, 58]. Therefore, a similar pressure level might exist for compression tights that reduce foot rotation angles. Future studies may systematically investigate the effect of pressure on foot rotation angles.

Fourth, we did not examine the long-term effects of the tights and the retention period of the modified angles after removing the tights. Future studies may consider investigating the long-term effects and retention time to address the utility of prolonged wear and the potential of these tights as a training tool for the persistent modification of foot rotation angles. Fifth, in compliance with the ethical requirements, the participants were informed of the aims of the study. The participants also could see and sense the taping lines during walking. The fact that the aim and conditions of the study were not blinded might introduce a possible bias toward the tights. Sixth, we attached markers on tights; the measure of the lower limb kinematics might be affected by the possible relative movement between the skin and the tights. However, the measurement of the most important output variable, the foot rotation angle, did not rely on the use of markers; we used pressure pad to measure that. Therefore, this limitation cannot crucially affect our main results. Finally, we could not clarify the clinical implications of the proposed compression tights for mitigating out-toeing gait. To the best of our knowledge, no study has assessed the minimal clinically important difference (MCID) for the foot rotation angles for dancers during walking. Hence, a future study may consider developing this MCID criterion to assess clinical implications for interventions designed to mitigate the out-toeing gait of dancers.

## Conclusion

We propose an effective, comfortable, and practical wearable means for modifying foot rotation angles, and verified its feasibility by demonstrating the short-term efficacy of the proposed compression tights in mitigating the out-toeing gait of female dancers. The high functionality and usability reported by the participants further support the feasibility of the proposed compression tights.

## Supporting information

S1 File Manuscript data. This file includes the obtained data of the foot rotation angles, and ratings reported by the participants for all criteria of perceived functionality and usability.
(XLSX)

S2 File This file includes three supplementary tables, five supplementary figures and one supplementary result. (DOCX)

## References

[1] Jenkyn TR, Hunt MA, Jones IC, Giffin JR, Birmingham TB. Toe-out gait in patients with knee osteoarthritis partially transforms external knee adduction moment into flexion moment during early stance phase of gait: a tri-planar kinetic mechanism. J Biomech. 2008;41(2):276–83. doi: 10.1016/j.jbiomech.2007.09.015 18061197

[2] Cao LA, Rethlefsen SA, Wren TA, Kay RM. Causes of out-toeing gait in children with cerebral palsy. Gait Posture. 2020;76:141–5. doi: 10.1016/j.gaitpost.2019.12.002 31855804

[3] Lincoln TL, Suen PW. Common rotational variations in children. J Am Acad Orthop Surg. 2003;11(5):312–20. doi: 10.5435/00124635-200309000-00004 14565753

[4] Macintyre J, Joy E. Foot and ankle injuries in dance. Clin Sports Med. 2000;19(2):351–68. doi: 10.1016/s0278-5919(05)70208-8 10740764

[5] Nourai MH, Fadaei B, Rizi AM. In-toeing and out-toeing gait conservative treatment; hip anteversion and retroversion: 10-year follow-up. J Res Med Sci. 2015;20(11):1084–7. doi: 10.4103/1735-1995.172833 26941813 PMC4755096

[6] Fabry G, Cheng LX, Molenaers G. Normal and abnormal torsional development in children. Clin Orthop Relat Res. 1994;302:22–6. 8168306

[7] Cimelli SN, Curran SA. Influence of turnout on foot posture and its relationship to overuse musculoskeletal injury in professional contemporary dancers: a preliminary investigation. J Am Podiatr Med Assoc. 2012;102(1):25–33. doi: 10.7547/1020025 22232318

[8] Chang A, Hurwitz D, Dunlop D, Song J, Cahue S, Hayes K, et al. The relationship between toe-out angle during gait and progression of medial tibiofemoral osteoarthritis. Ann Rheum Dis. 2007;66(10):1271–5. doi: 10.1136/ard.2006.062927 17267516 PMC1994298

[9] Uden H, Kumar S. Non-surgical management of a pediatric “intoed” gait pattern–a systematic review of the current best evidence. J Multidiscip Healthc. 2012;5:27. doi: 10.2147/JMDH.S28669 22328828 PMC3273377

[10] Khalaj N, Abu Osman NA, Mokhtar AH, Mehdikhani M, Wan Abas WA. Effect of exercise and gait retraining on knee adduction moment in people with knee osteoarthritis. Proc Inst Mech Eng H. 2014;228(2):190–9. doi: 10.1177/0954411914521155 24458100

[11] Richards R, van den Noort J, Van der Esch M, Booij M, Harlaar J. Gait retraining using real-time feedback in patients with medial knee osteoarthritis: Feasibility and effects of a six-week gait training program. Knee. 2018;25(5):814–24. doi: 10.1016/j.knee.2018.05.014 29933935

[12] Shih YC, Chau MM, Arendt EA, Novacheck TF. Measuring lower extremity rotational alignment: a review of methods and case studies of clinical applications. J Bone Joint Surg. 2020;102(4):343–56. doi: 10.2106/JBJS.18.01115 31743239

[13] Khan SJ, Khan SS, Usman J, Mokhtar AH, Abu Osman NA. Combined effects of knee brace, laterally wedged insoles, and toe-out gait on knee adduction moment and fall risk in moderate medial knee osteoarthritis patients. Prosthet Orthot Int. 2019;43(2):148–57. doi: 10.1177/0309364618796849 30192706

[14] Reeves ND, Bowling FL. Conservative biomechanical strategies for knee osteoarthritis. Nat Rev Rheumatol. 2011;7(2):113–22. doi: 10.1038/nrrheum.2010.212 21289615

[15] Xia H, Charlton JM, Shull PB, Hunt MA. Portable, automated foot progression angle gait modification via a proof-of-concept haptic feedback-sensorized shoe. J Biomech. 2020;107:109789. doi: 10.1016/j.jbiomech.2020.109789 32321637

[16] Bennell KL, Goldie PA. The differential effects of external ankle support on postural control. J Orthop Sports Phys Ther. 1994;20(6):287–95. doi: 10.2519/jospt.1994.20.6.287 7849748

[17] Shinohara M. Effects of prolonged vibration on motor unit activity and motor performance. Med Sci Sports Exerc. 2005;37(12):2120–5. doi: 10.1249/01.mss.0000178106.68569.7e 16331139

[18] Adamo DE, Martin BJ, Johnson PW. Vibration-induced muscle fatigue, a possible contribution to musculoskeletal injury. Eur J Appl Physiol. 2002;88(1):134–40. doi: 10.1007/s00421-002-0660-y 12436281

[19] Cui W, Wang C, Chen W, Guo Y, Jia Y, Du W, et al. Effects of toe-out and toe-in gaits on lower-extremity kinematics, dynamics, and electromyography. Appl Sci. 2019;9(23):5245–67.

[20] Presedo A, Simon A-L, Mallet C, Ilharreborde B, Mazda K, Pennecot G-F. Correlation between transverse plan kinematics and foot progression angle in children with spastic diplegia. J Pediatr Orthop B. 2017;26(3):211–6. doi: 10.1097/BPB.0000000000000416 27902635

[21] Song CY, Huang HY, Chen SC, Lin JJ, Chang AH. Effects of femoral rotational taping on pain, lower extremity kinematics, and muscle activation in female patients with patellofemoral pain. J Sports Sci Med. 2015;18(4):388–93. doi: 10.1016/j.jsams.2014.07.009 25127530

[22] Song CY, Lin JJ, Chang AH. Effects of femoral rotational taping on dynamic postural stability in female patients with patellofemoral pain. Clin J Sport Med. 2017;27(5):438–43. doi: 10.1097/JSM.0000000000000392 28036322

[23] Mikołajewska E. Side effects of kinesiotaping–own observations. J Health Sci. 2011;1(4):93–9.

[24] Kurz E, Anders C. Effects of wearing lower leg compression sleeves on locomotion economy. J Sports Sci. 2018;36(18):2105–10. doi: 10.1080/02640414.2018.1439355 29447545

[25] Bringard A, Perrey S, Belluye N. Aerobic energy cost and sensation responses during submaximal running exercise-positive effects of wearing compression tights. Int J Sports Med. 2006;27(05):373–8. doi: 10.1055/s-2005-865718 16729379

[26] Broatch JR, Brophy-Williams N, Phillips EJ, O’BRYAN SJ, Halson SL, Barnes S, et al. Compression garments reduce muscle movement and activation during submaximal running. Med Sci Sports Exerc. 2020;52(3):685–95. doi: 10.1249/MSS.0000000000002182 31592978 PMC7034367

[27] Hintzy F, Gregoire N, Samozino P, Chiementin X, Bertucci W, Rossi J. Effect of thigh-compression shorts on muscle activity and soft-tissue vibration during cycling. J Strength Cond Res. 2019;33(8):2145–52. doi: 10.1519/JSC.0000000000002402 31344011

[28] Doan B, Kwon YH, Newton R, Shim J, Popper E, Rogers R, et al. Evaluation of a lower-body compression garment. J Sports Sci. 2003;21(8):601–10. doi: 10.1080/0264041031000101971 12875311

[29] Kraemer WJ, Bush JA, Newton RU, Duncan ND, Volek JS, Denegar CR, et al. Influence of a compression garment on repetitive power output production before and after different types of muscle fatigue. Res Sports Med. 1998;8(2):163–84.

[30] Chan V, Duffield R, Watsford M. The effects of compression garments on performance of prolonged manual-labour exercise and recovery. Appl Physiol Nutr Metab. 2016;41(2):125–32. doi: 10.1139/apnm-2015-0335 26778138

[31] Lu L, Megahed FM, Cavuoto LA. Interventions to mitigate fatigue induced by physical work: A systematic review of research quality and levels of evidence for intervention efficacy. Hum Factors. 2021;63(1):151–91. doi: 10.1177/0018720819876141 31596613

[32] Hackman S, Dyson R, Abraham C, editors. Foot alignment and unipodal postural stability of dancers trained in classical ballet. ISBS-Conference Proceedings Archive; 2004.

[33] Sawilowsky SS. New effect size rules of thumb. J Mod Appl Stat Meth. 2009;8(2):26.

[34] Faul F, Erdfelder E, Buchner A, Lang A-G. Statistical power analyses using G* Power 3.1: Tests for correlation and regression analyses. Behav Res Methods. 2009;41(4):1149–60. doi: 10.3758/BRM.41.4.1149 19897823

[35] KS K 0051: *Sizing systems for female adult’s garments*. Eumseong. Korean Agency for Technology and Standards; 2019.

[36] ISO 8559–1. *Size designation of clothes—part 1*: *anthropometric definitions for body measurement*. Geneva. International Organization for Standardization 2017.

[37] van Melick N, Meddeler BM, Hoogeboom TJ, Nijhuis-van der Sanden MW, van Cingel RE. How to determine leg dominance: The agreement between self-reported and observed performance in healthy adults. PloS One. 2017;12(12):e0189876. doi: 10.1371/journal.pone.0189876 29287067 PMC5747428

[38] Armstrong HJ. Patternmaking for fashion design. 4 ed. Upper Saddle River, NJ: Pearson Prentice Hall; 2006.

[39] ISO 9241–11. *Ergonomics of human-system interaction—Part 11*: *Usability*: *Definitions and concepts*. Geneva. International Organization for Standardization; 2018.

[40] Bye E, McKinney E. Fit analysis using live and 3D scan models. Int J Cloth Sci Technol. 2010;22(2):88–100.

[41] Kim NY, Lee H. Influence of clothing pressure on blood flow and subjective sensibility of commercial sports compression wear. Cloth Text Res J. 2019;21(4):459–67.

[42] Park JH, Chun J. Comparison of evaluation methods for measuring pressure of compressionwear. Res J Costume Cult. 2013;21(4):535–45.

[43] Choi J, Kim N, Wu Y, Hong K. Effects of 3D compression suits on EEG analysis during and after walking. J Korean Soc Cloth Text. 2014;38(4):440–54.

[44] Heinemann AW, Bode R, O’reilly C. Development and measurement properties of the Orthotics and Prosthetics Users’ Survey (OPUS): a comprehensive set of clinical outcome instruments. Prosthet Orthot Int. 2003;27(3):191–206. doi: 10.1080/03093640308726682 14727700

[45] Demers L, Weiss-Lambrou R, Ska B. The Quebec User Evaluation of Satisfaction with Assistive Technology (QUEST 2.0): an overview and recent progress. Technol Disabil. 2002;14(3):101–5.

[46] Pathak P, Ahn J. A pressure-pad-embedded treadmill yields time-dependent errors in estimating ground reaction force during walking. Sensors. 2021;21(16):5511. doi: 10.3390/s21165511 34450953 PMC8401449

[47] Pathak P, Moon J, Roh S-g, Roh C, Shim, Ahn J. Application of vibration to the soles reduces minimum toe clearance variability during walking. PloS One. 2022;17(1):e0261732. doi: 10.1371/journal.pone.0261732 34982783 PMC8726470

[48] Norton KI. Standards for anthropometry assessment. Kinanthropometry and exercise physiology. 2018;4:68–137.

[49] Meyer C, Killeen T, Easthope CS, Curt A, Bolliger M, Linnebank M, et al. Familiarization with treadmill walking: How much is enough? Sci Rep. 2019;9(1):1–10. 30914746 10.1038/s41598-019-41721-0 PMC6435738

[50] Nüesch C, Overberg J-A, Schwameder H, Pagenstert G, Mündermann A. Repeatability of spatiotemporal, plantar pressure and force parameters during treadmill walking and running. Gait Posture. 2018;62:117–23. doi: 10.1016/j.gaitpost.2018.03.017 29547791

[51] Hamilton D, Aronsen P, Løken J, Berg I, Skotheim R, Hopper D, et al. Dance training intensity at 11–14 years is associated with femoral torsion in classical ballet dancers. Br J Sports Med. 2006;40(4):299–303. doi: 10.1136/bjsm.2005.020941 16556782 PMC2577517

[52] Hamilton WG, Hamilton LH, Marshall P, Molnar M. A profile of the musculoskeletal characteristics of elite professional ballet dancers. Am J Sports Med. 1992;20(3):267–73. doi: 10.1177/036354659202000306 1636856

[53] Khoo-Summers LC, Prather H, Hunt DM, Van Dillen LR. Predictors of first position turnout in collegiate dancers: the role of tibiofemoral external rotation and hip external rotation. Am J Phys Med Rehabil. 2013;92(2):136–42. doi: 10.1097/PHM.0b013e3182465dff 22286892

[54] Thielen M, Waible D, Krautwurst BK, Wolf SI, Dreher T. Effects of artificially induced bilateral internal rotation gait on gait kinematics and kinetics. Gait Posture. 2022;95:204–9. doi: 10.1016/j.gaitpost.2022.05.003 35533614

[55] ASTMD2594-04: Standard Test Method for Stretch Properties of Knitted Fabrics Having Low Power. ASTM International; 2016.

[56] Macintyre L. Designing pressure garments capable of exerting specific pressures on limbs. Burns. 2007;33(5):579–86. doi: 10.1016/j.burns.2006.10.004 17482762

[57] Mizuno S, Arai M, Todoko F, Yamada E, Goto K. Wearing lower-body compression garment with medium pressure impaired exercise-induced performance decrement during prolonged running. PloS One. 2017;12(5):e0178620. doi: 10.1371/journal.pone.0178620 28562650 PMC5451085

[58] Hill J, Howatson G, Van Someren K, Gaze D, Legg H, Lineham J, et al. The effects of compression-garment pressure on recovery after strenuous exercise. Int J Sports Physiol Perform. 2017;12(8):1078–84. doi: 10.1123/ijspp.2016-0380 28051341

