# Supplementary material for: Feasibility of mitigating out-toeing gait using compression tights with inward-directing taping lines
Source: PLoS One. 2023 Sep 21;18(9):e0291914. doi: 10.1371/journal.pone.0291914 (PMC10513335; doi:10.1371/journal.pone.0291914)
Supplement: S2 File — (DOCX) [file pone.0291914.s002.docx]

**Supplementary Information**

**S1 Table. Criteria and questions included in the user questionnaire.**

| **Section** | **Criteria** | | **Questions** |
| --- | --- | --- | --- |
| **Perceived**  **functionality** | Compression strength | | Did you feel excessive compression? |
|  | Directional tension strength | | Did you feel excessive tension with direction? |
| **Perceived**  **usability** | Satisfaction | Size | Were the tights worn appropriately? |
|  |  | Compression | Were you satisfied with the compression of the tights? |
|  |  | Product | Were you satisfied with the product? |
|  | Movement easiness | | Was it easy to walk? |
|  | Usefulness for gait correction | | Have you had positive changes in your ability to walk? |


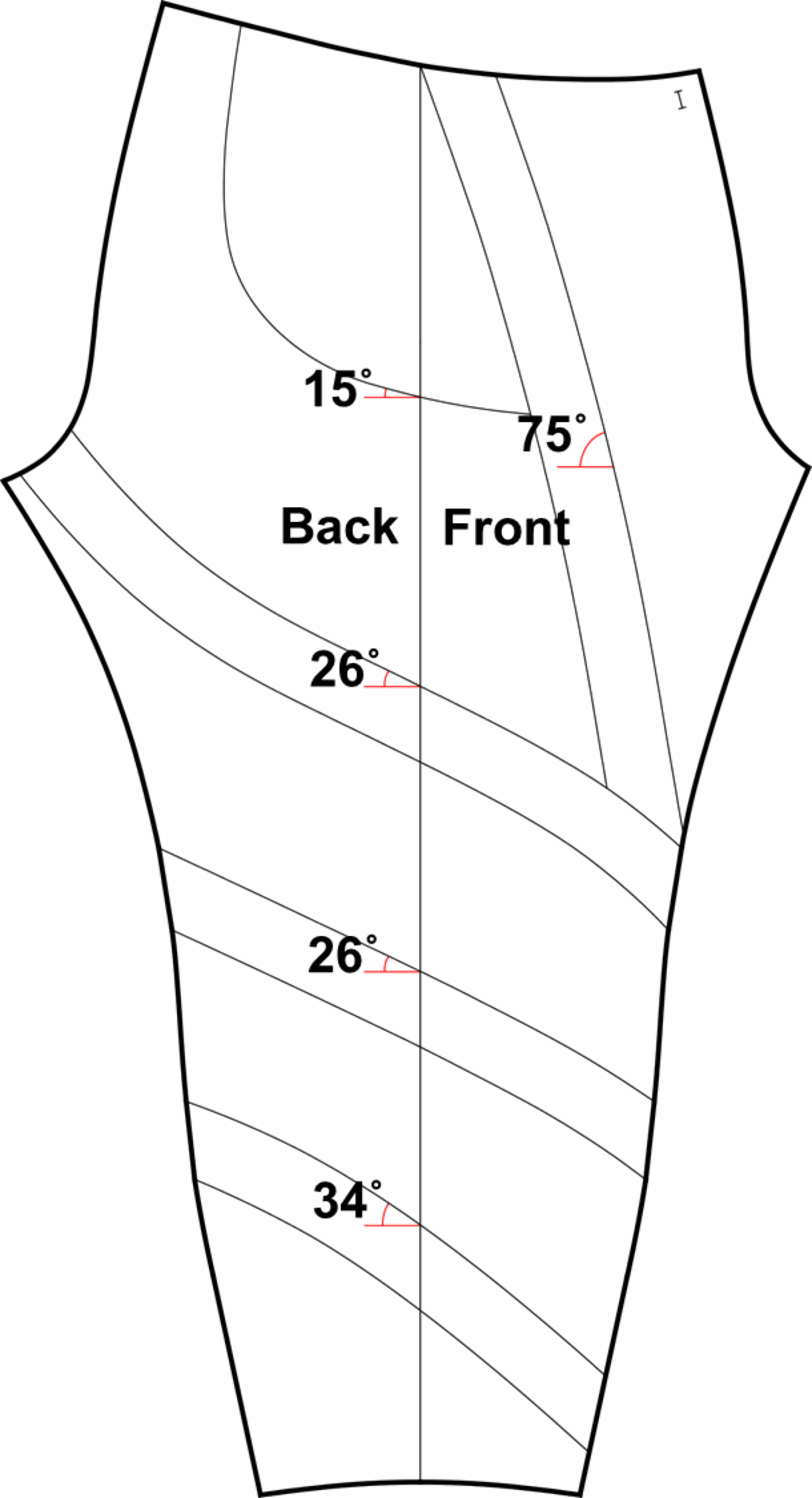


**S1 Fig. Pattern of the ICtights.**


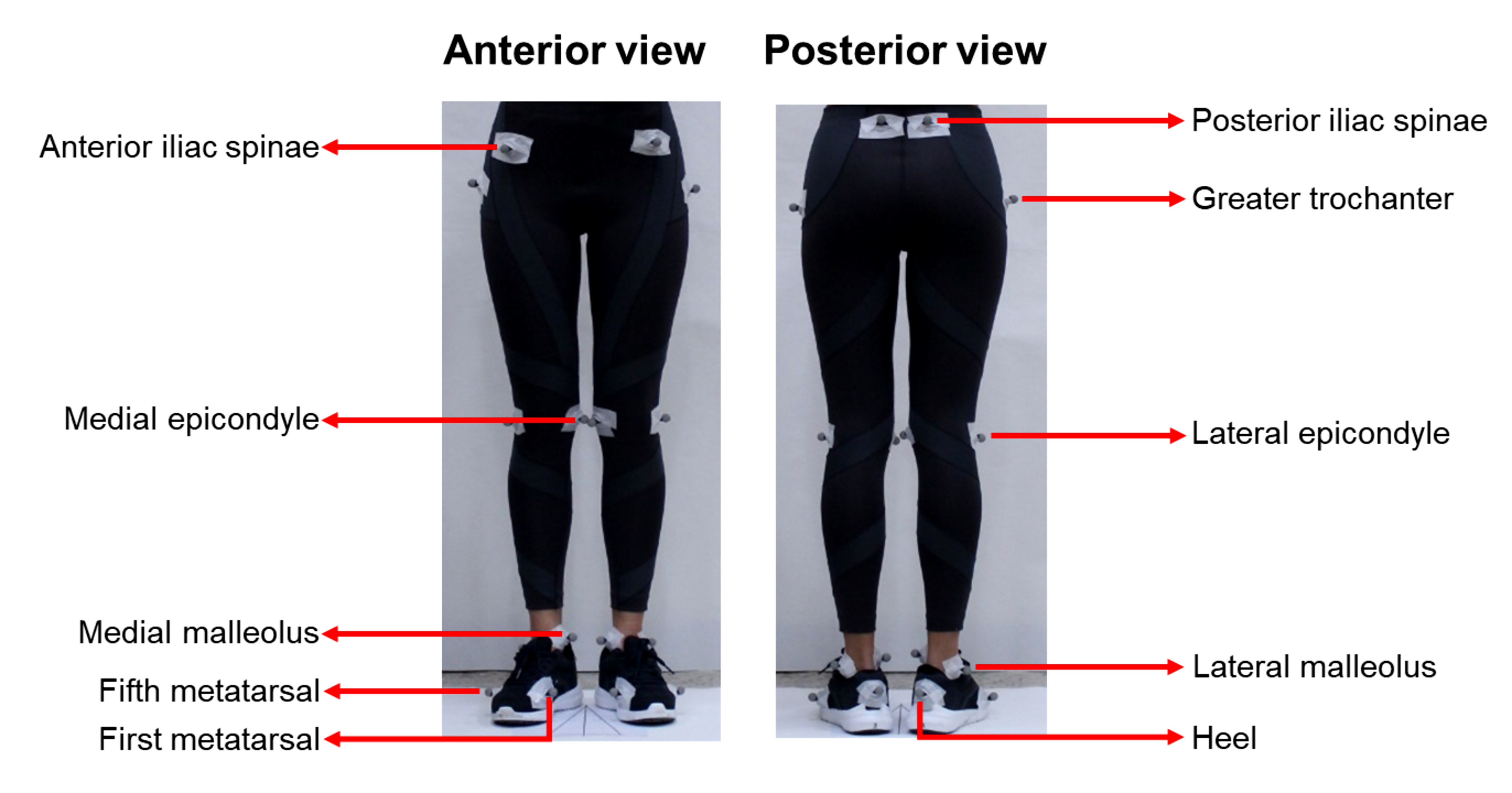


**S2 Fig. Illustration of the attachment positions of 20 retro-reflective markers on the anatomical landmarks of the lower limbs.** The markers were attached at the left and right heel, first and fifth metatarsal, medial and lateral malleolus, medial and lateral epicondyle, greater trochanter, and anterior and posterior iliac spinae


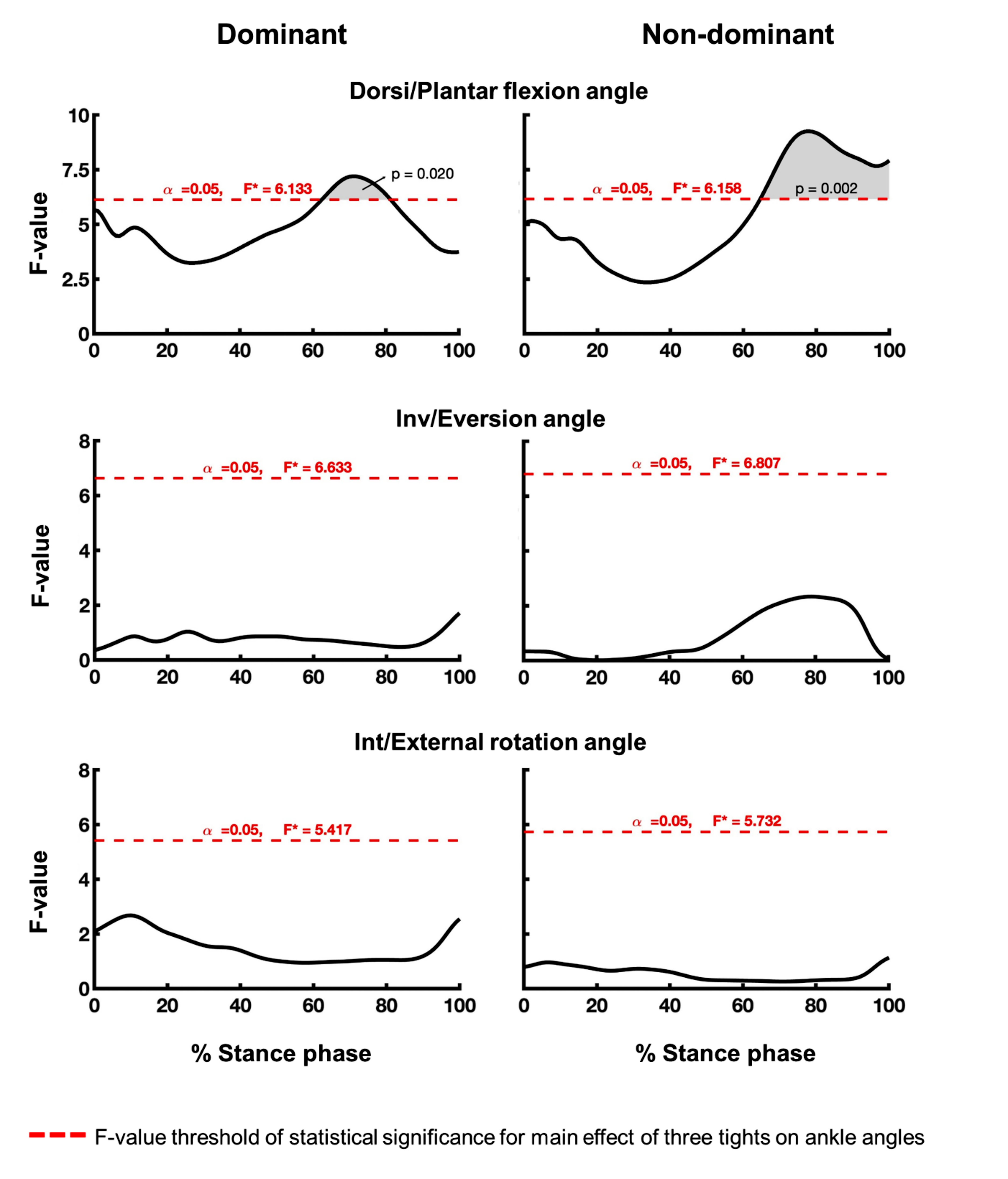


**S3 Fig. F-values of the main effects of tights on the three-dimensional ankle angles during the stance phase acquired using one-way repeated measures ANOVA.** The stance intervals were normalized between 0 and 100% (101 points), and ANOVA was performed using the SPM analysis. The black lines are the changes in F-values during the stance phase, and the dotted red line is the threshold for statistical significance for the main effect of tights on ankle angles.


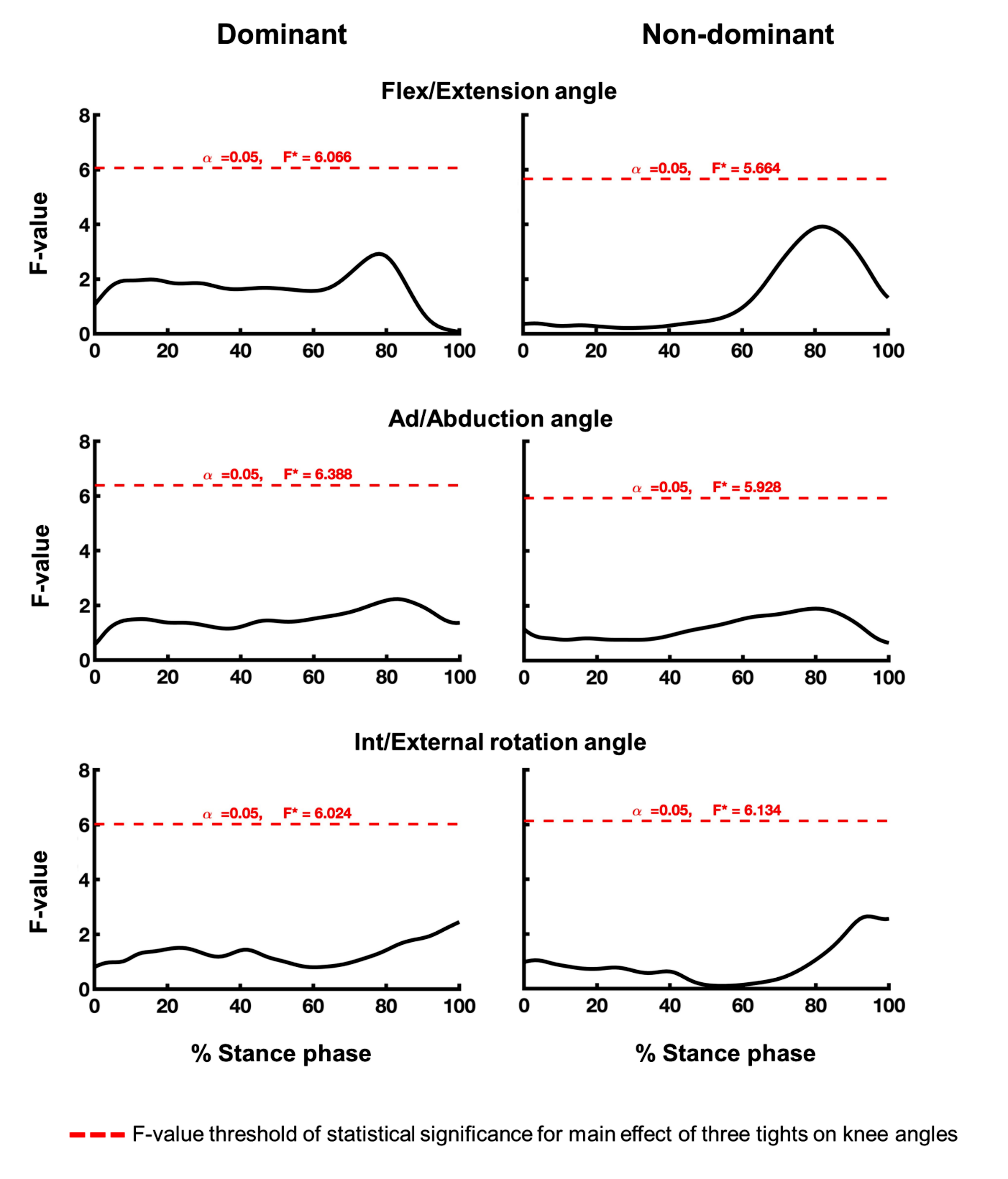


**S4 Fig. F-values of the main effects of tights on the three-dimensional knee angles during the stance phase acquired using one-way repeated measures ANOVA.** The stance intervals were normalized between 0 and 100% (101 points), and ANOVA was performed using the SPM analysis. The black lines are the changes in F-values during the stance phase, and the dotted red line is the threshold for statistical significance for the main effect of tights on knee angles.


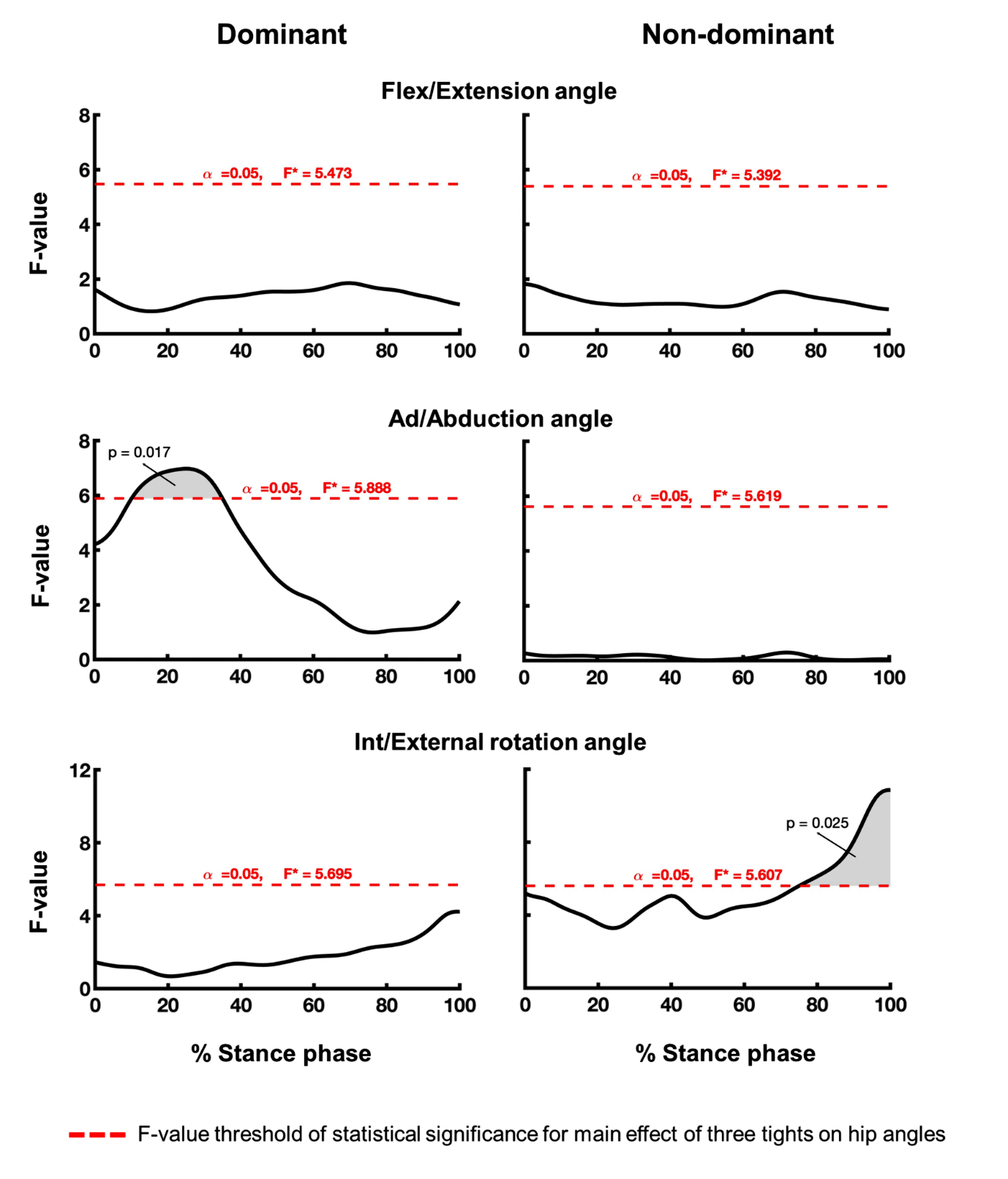


**S5 Fig. F-values of the main effects of tights on the three-dimensional hip angles during the stance phase acquired using one-way repeated measures ANOVA.** The stance intervals were normalized between 0 and 100% (101 points), and ANOVA was performed using the SPM analysis. The black lines are the changes in F-values during the stance phase, and the dotted red line is the threshold for statistical significance for the main effect of tights on hip angles.

**S2 Table. Results of the Shapiro–Wilk test assessing the normality of perceived functionality and usability ratings.**

| **Section** | **Criteria** | **Normtights** | **ICtights** |
| --- | --- | --- | --- |
| **Perceived functionality** | Perceived functionality | W(12) = 0.868,  p = 0.062 | W(12) = 0.926,  p = 0.339 |
|  | Compression strength | W(12) = 0.916,  p = 0.257 | W(12) = 0.956,  p = 0.721 |
|  | Directional tension strength | **W(12) = 0.738,**  **p = 0.002** | W(12) = 0.919,  p = 0.279 |
| **Perceived usability** | Perceived usability | W(12) = 0.980,  p = 0.982 | W(12) = 0.964,  p = 0.843 |
|  | Satisfaction | W(12) = 0.917,  p = 0.263 | W(12) = 0.940,  p = 0.492 |
|  | Size satisfaction | W(12) = 0.888,  p = 0.110 | **W(12) = 0.808,**  **p = 0.012** |
|  | Compression satisfaction | W(12) = 0.890,  p = 0.117 | W(12) = 0.901,  p = 0.164 |
|  | Product satisfaction | W(12) = 0.914,  p = 0.242 | **W(12) = 0.828,**  **p = 0.020** |
|  | Movement easiness | W(12) = 0.898,  p = 0.149 | W(12) = 0.909,  p = 0.208 |
|  | Usefulness for gait correction | **W(12) = 0.850,**  **p = 0.037** | **W(12) = 0.784,**  **p = 0.006** |

**S3 Table. Results of paired t-test and Wilcoxon-signed rank test comparing the perceived functionality and usability of the Normtights and ICtights.**

|  | **Criteria** | **Paired t- test**  **results** | **Criteria** | **Wilcoxon-signed**  **rank test results** |
| --- | --- | --- | --- | --- |
| **Perceived functionality** | Perceived functionality | **t(11) = 5.411,**  **p < 0.001,**  **ES = 2.207** | Directional tension strength | **Z(11) = 2.869,**  **p = 0.004,**  **ES = 0.828** |
|  | Compression strength | **t(11) = 5.968,**  **p < 0.001,**  **ES = 2.206** |  |  |
| **Perceived usability** | Perceived usability | **t(11) = 2.277,**  **p = 0.044,**  **ES = 1.049** | Size satisfaction | Z(11) = 0.153,  p = 0.878,  ES = 0.044 |
|  | Satisfaction | t(11) = 1.444,  p = 0.177,  ES = 0.670 | Product satisfaction | Z(11) = 1.924,  p = 0.054,  ES = 0.555 |
|  | Compression satisfaction | t(11) = 2.107,  p = 0.059,  ES = 0.851 | Usefulness for gait correction | **Z(11) = 2.958,**  **p = 0.003,**  **ES = 0.854** |
|  | Movement easiness | t(11) = 0.158,  p = 0.877,  ES = 0.076 |  |  |

**S1 Results. Supplementary comments from the participants.**

The participants’ supplementary comments supported the quantitative survey results in three aspects. First, the participants felt compression and directional tension, but they were satisfied with the compression level and size of the ICtights.

*Participant 5: “The pressure on the waist was moderate and the pressure on the thighs and calves was high.”*

*Participant 8: “I liked the tightness of these tights (ICtights).”*

*Participant 12: “I felt my thighs and knees curled inward and muscle tension. I felt the ankles grabbed me inwards.”*

Second, the ICtights did not significantly restrict movement despite the relatively high compression.

*Participant 4: “It was easy to walk by holding the muscles, and I felt light in my thighs, especially when walking.”*

Third, the participants perceived ICtights to be useful in gait correction.

*Participant 3: “I am satisfied because I felt a correction when I walked.”*

*Participant 9: “These tights (ICtights) gave strength to my legs and lower abdomen, and I felt that I was able to stand upright and walk. There was a feeling that the legs were gathered inward and that the feet were gathered.”*
